# Chloride Diffusion in Concrete Made with Coal Fly Ash Ternary and Ground Granulated Blast-Furnace Slag Portland Cements

**DOI:** 10.3390/ma15248914

**Published:** 2022-12-13

**Authors:** Miguel Ángel Sanjuán, Rosa Abnelia Rivera, Domingo Alfonso Martín, Esteban Estévez

**Affiliations:** 1 Department of Science and Technology of Building Materials, Civil Engineering School, Technical University of Madrid, 28040 Madrid, Spain; 2 Department of Cement Chemical Testing, LOEMCO, Technical University of Madrid (UPM), Getafe, 28906 Madrid, Spain; 3 Mine and Energy Engineering School, Technical University of Madrid (UPM), 28003 Madrid, Spain

**Keywords:** cement, concrete, recycled materials, coal fly ash, granulated blast-furnace slag, durability, chloride diffusion

## Abstract

Ternary Portland cement usage with a high amount of cement constituents different from clinker can afford great climate change advantages by lowering the Portland cement clinker content in the final product. This will contribute to cutting greenhouse gas emissions to close to zero by 2050. Such ternary Portland cements can be composed of different amounts of ground granulated blast-furnace slag (GBFS), coal fly ash (CFA), and clinker (K). Cements made with GGBFS, or CFA boast pozzolanic characteristics. Therefore, they would improve both the concrete compressive strength at later ages and durability. The 28- and 90-days mechanical strength test, non-steady state chloride migration test, described in NT BUILD 492, and natural chloride diffusion test (NT BUILD 443) were performed in concrete. Ternary cements made with GBFS and/or CFA presented better chloride diffusion resistance than concrete made with plain Portland cements. Furthermore, the development of compressive strength was delayed. The service life study was developed for concretes made with ternary cements with regard to the chloride penetration case.

## 1. Introduction

The rapid growth of populations and cities has led to significantly increasing demand for Portland cement. This phenomenon is particularly harmful as it can increase the risk of more severe climate change impacts. Currently, Portland cement production accounts for approximately 7.4% of the anthropogenic emissions of carbon dioxide across the world (2016: 2.9 Gtons of carbon dioxide emissions) [1], i.e., Portland cement is the second largest emission production sector after steel.

Accordingly, a reduction in carbon dioxide emissions within the cement manufacturing sector can be achieved by lowering the amount of clinker in blended cements [2]. Some industrial wastes containing principally inorganic constituents, which may contain metals and organic materials such as ground granulated blast-furnace slag (GGBFS) and coal fly ash (FA), have recently gained relevance as sustainable and durable Portland cement constituents because of their environmental benefits [3].

In addition, mechanical strength improvement was found in some ternary systems made with FA and GGBFS [4,5].

Compressive strength development is particularly enhanced with very fine granulated blast-furnace slag and coal fly ash [6]. In contrast, non-enhancement in the mechanical strength in other ternary mixes has also been reported [7]. Accordingly, it can be concluded that the final properties of such ternary systems are reached as result of the mixture of individual properties of all the cement components and their synergy [8].

Global production of ground granulated blast-furnace slag is expected to reach 280 million tons in 2025, with China’s production to total around 153 million tons [9,10]. The European production was 24.6 million tons in 2016 [11], while the American production was 17.0 million tons in 2019 [12]. Worldwide consumption of GGBFS is projected to achieve about 269 million tons in 2025. China is the largest consumer of slag (2020), but its share is expected to decline from 62% to 55% (2025), as China will reduce steel production.

The widespread application of coal fly ash (FA) and ground granulated blast-furnace slag (GGBFS) ternary Portland cements in building and civil engineering would have significant positive impacts on the environment, as the cement sector is a major emitter of greenhouse gases [2]. Accordingly, there is an urgent need to assess the durable performance of concrete incorporating these ternary Portland cements. In particular, chloride ion penetration into reinforced concrete and the resulting microcracking due to the reinforcement chloride-induced corrosion is a major concern for its durability. Thus, assessment of the durability of ternary Portland cement concrete from its chloride permeation properties is a key issue to ensure the long-term service life of reinforced concrete structures, most of all when reinforced concrete is utilized in chloride ion-rich environments, such as new structures built in settlements in coastal and marine ecosystems. The novelty of this paper relies on the comparison of ternary cements regarding their chloride ingress resistance.

### 1.1. Chloride Diffusion in Concrete Incorporating Ground Granulated Blast-Furnace Slag and Coal Fly Ash

The methods commonly used to determine the chloride diffusion coefficient are defined by the standards ASTM C 1556 [13,14], AASHTO T 259 [15] and NT BUILD 443 [16]. They are based on Fick’s 2nd law of diffusion and their main disadvantage is the testing duration. In contrast, in the ASTM C 1202 [17] and NT BUILD 492 [18] standards, the flow of chloride ions into concrete is accelerated by an electric field. This procedure speeds up the chloride ion flow throughout the concrete sample and the passed charge (ASTM C 1202) or the value of migration coefficient (NT BUILD 492) is measured.

Blast-furnace slag (ground granulated) and coal fly ash have been widely employed as constituents in common Portland cements since they enhance some properties of concrete by chemical and physical (filler, dilution, nucleation) effects, principally as a function of the effectiveness of the amount and particle size distribution (PSD) [19]. Furthermore, they exert a positive effect in preventing chloride ions, among other important ions, from penetrating into the concrete.

Some studies have reported the influence of coal fly ash addition on the chloride-resistance of Portland cement-based materials. In general, concrete made with Portland-fly ash cement or pozzolanic cement made with coal fly ash offer a higher resistance to chloride ingress than concretes made with CEM I [20].

The coal fly ash pozzolanic reaction directly affects the content of hydration products, which positively influences and determines the chloride resistance characteristics. Therefore, parameters influencing the pozzolanic reaction, such as water/cement ratio, replacement level of coal fly ash and type of coal fly ash, will play a key role [21,22]. Nevertheless, the mentioned parameters do not show a linear relationship with the chloride resistance of concrete and its binding capacity, i.e., when the coal fly ash replacement ratio is higher than 40%, the concrete chloride resistance will decrease [23]. The pozzolanic reaction and micro-filler effect provided by the coal fly ash particles promote stiffness in the cement paste matrix, which in turn generates a more durable material [24,25]. In contrast, the dilution effect of the limestone decreases the mechanical performance at later stages [26,27].

Ground granulated blast-furnace slag has been utilized for many years as a Portland cement constituent to improve the mechanical and durability characteristics of concrete [28]. Concrete incorporating cement with ground granulated blast-furnace slag presented higher resistance to chloride ion penetration than plain concrete [29].

This paper carried out non-steady state chloride migration test, described in NT BUILD 492, and a natural chloride diffusion test (NT BUILD 443) on the concrete samples with ternary cements, whose measurements were used to estimate the service life, which contributes in a major way to promoting the usage of industrial wastes as Portland cement constituents. One of the milestones of the novelty and relevance of this study is the determination, for the first time, of the chloride migration and diffusion coefficients of some new ternary cements covered by the European standard EN 197-5:2021 [30].

Furthermore, the challenge will be to move from traditional concrete uses to more widespread applications. Accordingly, experimentally determined results of chloride ingress coefficients determined from chloride migration tests are presented and discussed. Results from chloride diffusion tests carried out in accordance with standard NT BUILD 443, and results from non-steady-state chloride migration tests conducted in accordance with standard NT BUILD 492, are also compared.

### 1.2. Service Life Estimation

Traditionally, the relevance for safety of concrete structures has been strong, and its mechanical strength will be sufficiently guaranteed by a high safety factor. In contrast, the influence of the reinforced concrete structure degradation has not been considered systematically. Currently, architects and civil engineers are attempting to estimate the whole service life of reinforced concrete structures. This will certainly be hampered by many shortcomings, particularly as the exposure classes are usually not even well-defined and will likely change during the operational period of the reinforced concrete structures. The chloride-induced corrosion of steel reinforcement embedded in reinforced concrete structures is undoubtedly one of the most significant durability concerns [31]. For all the decisions, considerable knowledge on durability and service life of materials is needed. Furthermore, the information must be consistent.

In the operational phase of reinforced concrete structures, the most appropriate means of maintenance and replacement is planned. It is at this point that the information about the degradation effect on the service life must be as accurate as possible. A reinforced concrete structure is built for use for a certain length of a time, for at least 50 years. In addition, it is broadly acknowledged that about 80% of operation, maintenance and repair costs of a building are set in the 20% cost of the design process [32], i.e., many decisions that later affect the whole life cost of the reinforced concrete structure are taken during the design phase. Accordingly, great care must be taken to improve the material selection of the new reinforced concrete structures during the design phase.

## 2. Materials and Methods

### 2.1. Ternary Cement’s Constituents

Common ternary cements were made by mixing a CEM I 42.5 R—EN 197-1 [33], coal siliceous coal fly ash (V) and GGBFS. Their chemical characteristics are given in Table 1. In addition, the ground granulated blast-furnace slag reactive calcium oxide (CaO) and reactive silica (SiO_2_) amounts were 3.84% and 35.96%, respectively. Free lime contents in the coal fly ash and Portland cement were 0.5% and 1.31%, respectively.

The SO_3_, Al_2_O_3_, Fe_2_O_3_, CaO, SiO_2_, MgO, Na_2_O, K_2_O, insoluble residue (IR), loss on ignition (LOI), and Cl^−^ contents have been determined by the analytical methods described in EN 196-2 [34]. This standard specifies the methods for chemical analysis of cement. Furthermore, it defines the reference methods, as well as, in certain cases, alternative methods which can be considered to be equivalent.

Fineness was determined according to EN 196-6 [35] for the Portland cement (3246 cm^2^/g) and coal fly ash (3772 cm^2^/g). Their densities were 2.4 g/cm^3^ and 3.12 g/cm^3^, respectively. The granulated blast-furnace slag was ground until the desired degree of fineness is reached, 3489 cm^2^/g for SA and 4630 cm^2^/g for SB.

### 2.2. Cement Mix Design

Portland cements manufactured for this research were made up by uniting all the cement constituents, i.e., CEM I 42.5 R, siliceous fly ash (FA) and GGBFS in the proportions provided in Table 2. Accordingly, eight Portland cements were made in addition to the reference cement (100% CEM I 42.5 R–Holcim, Villaluenga, Spain). FA and GGBFS were provided by Cementos Tudela Veguín, S. A., Aboño, Spain.

### 2.3. Concrete Mix Design

Two concrete qualities (Table 3 and Table 4) have been made to evaluate the chloride resistance performance of the ternary cements shown in Table 2.

### 2.4. Methods

#### 2.4.1. Compressive Strength

Cylindrical specimens (∅15 × 30 cm) were made of concrete according to EN 12390-2:2019 [36]. They were cured under water and tested for 28- and 90-days compressive strength according to EN 12390-3:2019 [37]. The average compressive strength result determined from two results was taken.

#### 2.4.2. Chloride Penetration Profiles and Chloride Diffusion Coefficient Determination

**Figure 1:**
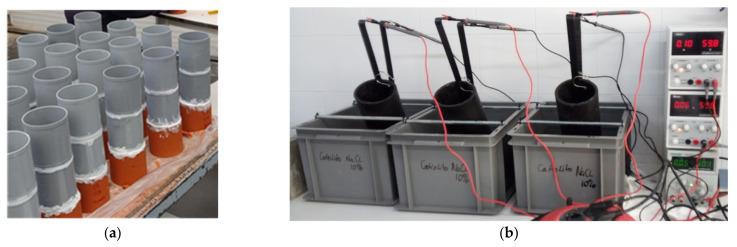
Chloride penetration methods: (**a**) NT BUILD 443 standard; (**b**) rapid chloride migration (RCM) test according to NT BUILD 492 standard.

The test to determine the chloride diffusion coefficient was carried out according to the method described in the NT BUILD 443 standard [16]. Four cylindrical samples of 100 mm diameter and 200 mm height were cast for each type of concrete and cement. Two test slices of 100 ± 1 mm thick were obtained from each 100 mm-diameter concrete specimen. Then, they were insulated on all surfaces of the concrete with epoxy resin, except the top area (Figure 1a). The orange zone in Figure 1a is the epoxy resin used to completely cover the concrete specimen. The white part is the silicon utilized to fix the grey pipes to the concrete specimen.

All the samples were soaked in limewater and then stored in a container. All faces of the concrete except the one to be exposed to the chloride solution were then dried at room temperature until constant weight was achieved. Later, the top of the concrete specimen was exposed to distilled water containing sodium chloride (30 ± 1 g/L NaCl–165 ± 1 g/L NaCl). This solution was replaced every 5 weeks with a new pure one. After specified exposure times of 3, 6, 9 and 12 months, thin layers were ground off parallel to the exposed surface of the concrete specimen and the chloride content of such layers, C_x_, was measured. The initial chloride content, C_0_, was determined at a proper depth below the exposed area to the chloride ions solution. The effective chloride transport coefficient, D_e_, and the boundary condition of the chloride profile at the exposed surface, C_s_, were calculated. The non-stationary diffusion coefficient, Dns, was determined according to the standard NT BUILD 443 [16], by fitting the chloride ions concentration profile acquired from the test to the integrated form of the Fick’s second law for semi-infinite media). This was carried out by using the related values of chloride concentration, C_x_, and depth below the exposed surface, x.

#### 2.4.3. Non-Steady State Chloride Migration Test—NT BUILD 492

In order to calculate the non-steady-state chloride migration coefficient of concrete the rapid chloride migration (RCM) test was performed according to NT BUILD 492.

First, cylindrical concrete specimens with Ø100 × 100 ± 1 mm (diameter × height) were cast and a Ø50 ± 1 mm thick slice was cut from the central part of the cylinder as the test sample. Then, the concrete specimens were kept in a desiccator and vacuumed at 1 kPa/min, thereby providing stable vacuum throughout concrete. They were kept in the desiccator for 4 h, and then, a saturated calcium hydroxide solution was added to ensure that all the specimens were immersed. After 1 h, air was entered in the desiccator to recover the atmospheric pressure. Twenty-four hours later, the concrete specimens were clamped, and the entire assembly was put into a test container. The end area was the one to be exposed to the saline solution containing chlorides (10% NaCl by mass, about 2 N. Catholyte). The anolyte contains a 0.3 N NaOH solution (about 12 g/L NaOH). 

Chloride ion transport was accelerated by the application of an electrical field. Concrete samples were located between the anode and cathode. In this arrangement, the cell consists of cathodes and anodes connected to each other and to the external 60 V DC voltage supply (i < 5 mA) at 25 °C (Figure 1b). The initial and final current through each specimen were recorded and the voltage can be adjusted throughout the test, if necessary. Therefore, the external electrical potential applied axially across the specimen forces the chloride ions to migrate into the specimen. 

After testing, the concrete specimen was axially split, and a 0.1 M silver nitrate solution was sprayed on the freshly split surface. The chloride ingress depth was measured from the formation of silver chloride, which accounts for the appearance of the white salt precipitation. Afterwards, this value of penetration depth is used to calculate the chloride migration coefficient from the non-steady state chloride migration test.

**Figure 2:**
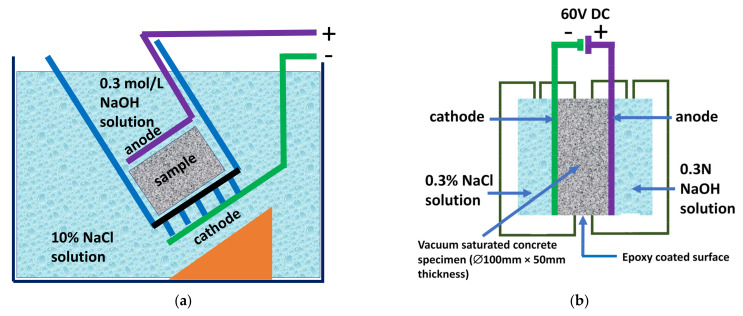
Accelerated Chloride Penetration Tests: (**a**) Schematic illustration of rapid chloride migration (RCM) experimental setup defined in the NT BUILD 492 standard; (**b**) Schematic illustration of Rapid Chloride Penetration Test (RCPT) according to ASTM C 1202 standard.

In the ASTM C 1202 [17] and NT BUILD 492 [18] standards, the flow of chloride ions into concrete is accelerated by an electric field. This procedure speeds up the chloride ions flow throughout the concrete sample and the passed charge (ASTM C 1202) or the result of the chloride migration coefficient (NT BUILD 492) is measured (Figure 2). Nevertheless, they can only be used for comparing protective characteristics of these concretes and not for any other purposes, such as service-life estimation. Apart from these two methods, the accelerated chloride migration test (ACMT) was also developed to measure both electrical current and accumulative chloride ions passing through the concrete specimen. As a result, a good correlation between both parameters was found [38].

## 3. Results and Discussion

### 3.1. Compressive Strength

**Figure 3:**
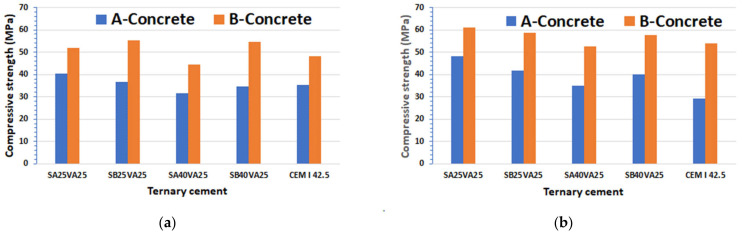
A-Concrete and B-Concrete compressive strength (MPa): (**a**) at 28-days; (**b**) at 90-days.

Mechanical strength of concretes A and B was assessed by compression strength testing at 28- and 90-days, which is the capacity of the concrete to withstand loads tending to reduce size (Figure 3). This parameter serves to assess whether concrete mix execution has been performed properly or not. Furthermore, the compressive strength test can afford a general view over other properties of the concrete.

According to the European standard EN 206 [39], the minimum characteristic cylindrical compressive strength at 28-days (f_ck_) for C30/37 concrete grade is 30 MPa, while for C45/55 concrete grade it is 45 MPa. Accordingly, Concrete A is categorized as grade C30/37 and Concrete B as grade C45/55. Ground granulated blast-furnace slag fineness has an effect more noticeable for concrete B than for concrete A (SA: 3489 cm^2^/g, SB: 4630 cm^2^/g).

With finer ground granulated blast-furnace slag, the compressive strength of concrete A is generally improved. Ternary cements SA25VA25 and SB25VA25, in both A and B concretes, improved the mechanical performance at 28 and 90 days in relation to the reference concrete made only with CEM I. This fact suggests a synergic effect of the FA and GGBFS [3].

**Figure 4:**
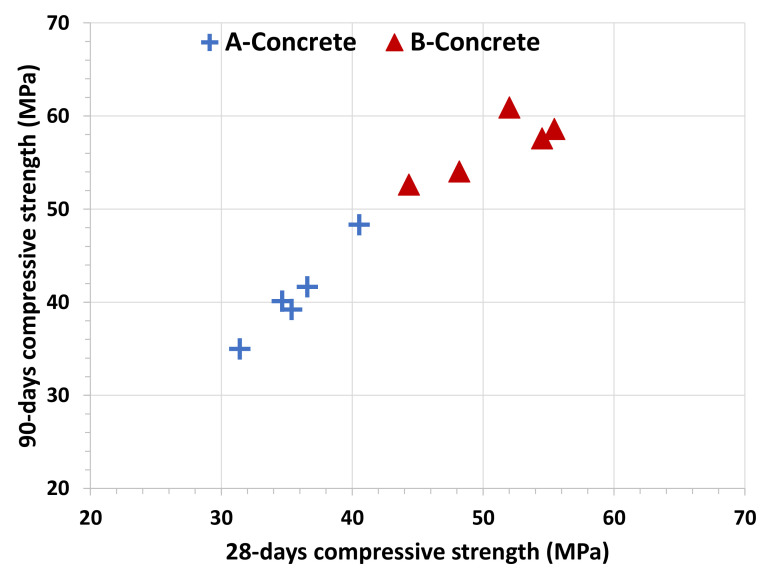
28-days versus 90-days compressive strength (MPa) relationship.

The relationship between the 28-days and 90-days compressive strength is shown in Figure 4. There was found a quasi-linear relationship between these two parameters for concrete A (R^2^ = 0.97). Equation (1) shows the mentioned relationship. In contrast, concrete B showed no correlation (R^2^ = 0.65).

90-days compressive strength = 1.45 × 28-days compressive strength − 10.79 (1)

Concrete B gains greater strengths with time and is denser than concrete A. However, the development of compressive strength of concrete B is more heterogenous and, consequently, achieving a good correlation between results obtained at 28 and 90 days is more difficult.

### 3.2. Chloride Diffusion Coefficient of Concrete

One experimental testing method has been used to determine the chloride diffusion coefficient based on Fick’s second law, which is concerned with concentration gradient changes with time. First, it is quite convenient to distinguish between the apparent chloride diffusion coefficient (D_app_), which is the value obtained in a non-steady-state test such as the 90-day ponding test, where chloride binding is occurring simultaneously with the diffusion process. In contrast, the effective diffusion coefficient (D_eff_) is attained when the chloride diffusion test is carried out under steady-state conditions in concrete previously saturated with chloride ions; accordingly, no chloride binding takes place during diffusion. Therefore, D_app_ is normally greater than D_eff_ since the chloride penetration front progresses throughout the concrete retarded by the binding. From the results recorded in the non-steady state chloride diffusion test defined in NT BUILD 443 [16], the effective chloride transport coefficient (D_eff_) for investigated concrete specimens is determined and summarized in Table 5. This test overcomes some of the shortcomings of the ponding test since the test specimens are saturated with lime water. Therefore, the initial sorption effect is prevented. In addition, all the surfaces are sealed except the top one, which is exposed to the chloride solution. The exposure time is 90 days.

**Figure 5:**
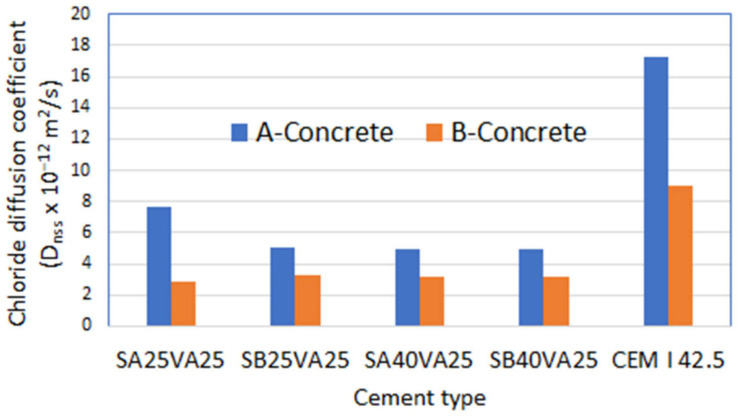
Chloride diffusion coefficient (D_eff_ × 10^−12^ m^2^/s) determined after 90 days of testing time.

As expected, concrete A was inferior to concrete B in resisting chloride diffusion, i.e., they have chloride diffusion coefficients of 17.3 × 10^−12^ m^2^/s and 8.99 × 10^−12^ m^2^/s, respectively. These results are in line with the higher chloride permeability measured in ternary cement concrete A compared to ternary cement concrete B (Figure 5). While similar natural chloride diffusion coefficients are found in concrete B made with FA and GGBFS (2.89 × 10^−12^ m^2^/s–3.36 × 10^−12^ m^2^/s), a wider range was measured in concretes A (4.98 × 10^−12^ m^2^/s–7.64 × 10^−12^ m^2^/s).

The performance of ternary cements added to concrete B in resisting chloride diffusion improves with the increase of ground granulated blast-furnace slag. This fact is more evident in concrete A.

No effect of GGBFS fineness on chloride diffusion coefficient was detected in cements with a high ground granulated blast-furnace slag content (40%). In contrast, with lesser amounts of ground granulated blast-furnace slag (25%), the natural chloride diffusion coefficient is lower for the concrete with the cement containing the coarser ground granulated blast-furnace slag (SB), but it is slightly higher for concrete B. This is probably due to the more efficient and effective synergic effect between the coal fly ash and the coarse ground granulated blast-furnace slag.

Frederiksen et al. found chloride migration coefficients higher than the chloride diffusion ones [40]. In contrast, we have obtained the opposite outcome. For concretes without Supplementary Cementitious Material, (SCM), a water/cement ratio of 0.70, and cement content of 265 kg/m^3^, they found a chloride diffusion coefficient with the immersion test of 35.50 × 10^−12^ m^2^/s, and a chloride migration coefficient of 40.25 × 10^−12^ m^2^/s [40]. For concrete A (w/c = 0.7, 250 kg/m^3^), we have determined lower values of chloride diffusion coefficient (17.3 × 10^−12^ m^2^/s) and chloride migration coefficient (2.97 × 10^−12^ m^2^/s). 

Conversely, they found lower coefficients with denser concretes (water/cement = 0.50 and 327 kg cement/m^3^ concrete) for the immersion test (16.15 × 10^−12^ m^2^/s) and migration test (18.55 × 10^−12^ m^2^/s) [40]. For concrete B (w/c = 0.5, 327 kg/m^3^), we have also determined lower values of the chloride diffusion coefficient (8.99 × 10^−12^ m^2^/s) and chloride migration coefficient (2.86 × 10^−12^ m^2^/s). In any event, it should be noted that the chloride diffusion coefficients of concretes A and B are in the same range as the ones found in the literature. Lower values of 3.10 × 10^−12^ m^2^/s [41] and 3.13 × 10^−12^ m^2^/s [42] have been reported with water/cement ratios of 0.30 and 0.40, and cement contents of 485 kg/m^3^ and 400 kg/m^3^, respectively.

The protective properties of concrete with steel reinforcement depend on the type used in the concrete mix design and it may be evaluated with a chloride diffusion coefficient obtained by using the NT BUILD 443 standard. This experimental procedure refers to the reinforced concrete exposed to saline solutions containing chlorides that are present mainly in deicing salt agents and seawater. Such adverse environments are complex systems, whereby some electrostatic interactions of chloride ions with other ions, such as calcium, sodium, potassium, among others, occur in the concrete pore solution. In most cases, electrostatic interactions are generally combined with other interactions, i.e., chloride ions can be adsorbed on the surface of the hydrated cement constituents and react with some aluminum-containing phases present in the cement. Nevertheless, Fick’s second law can be utilized to model the chloride ion flow through the concrete regardless of the combined physical, chemical, and electrostatic processes involved in the chloride ion ingress. 

For instance, following the method defined by the NT BUILD 443 standard. The major disadvantage of this method is the length of the test. In contrast, an electric field can be applied to speed up the flow of chloride ions and measure the charge passing through the concrete or calculate the migration coefficient.

### 3.3. Non-Steady-State Chloride Migration Coefficient of Concrete

**Figure 6:**
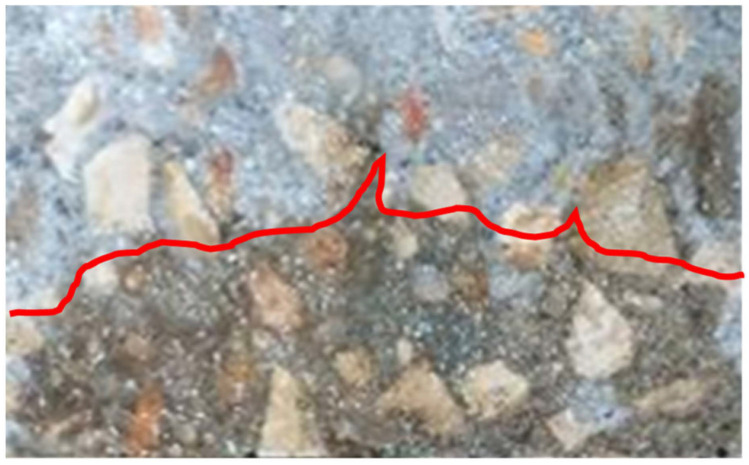
Specimens split for chloride penetration depth measurement after 96 h at 60 V.

Once the rapid chloride migration (RCM) experimental test (which is defined in the NT BUILD 492 standard) has been terminated, the concrete specimens were axially split up into two halves in such a way as to allow the 0.1 M AgNO_3_ solution to be sprayed on the fracture surface to measure the chloride penetration depth (Figure 6). Such a chloride penetration depth was considered to calculate the non-steady-state migration coefficient (D_nssm_).

**Figure 7:**
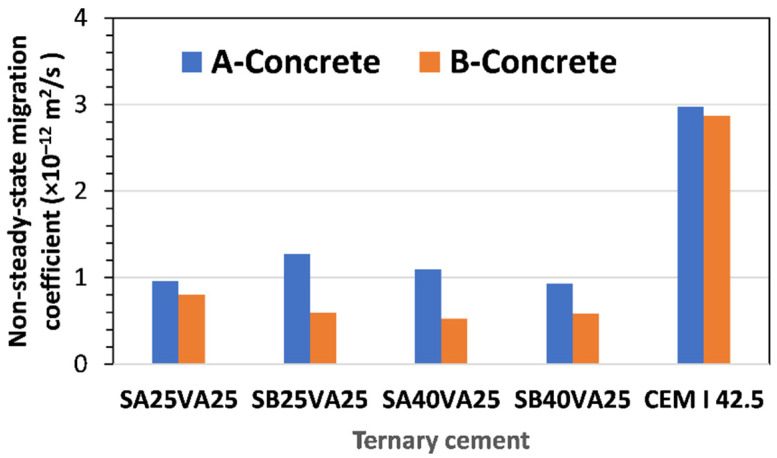
Non-steady-state migration coefficient (D_nssm_ ×10^−12^ m^2^/s) determined according to the NT BUILD 492 standard.

Non-steady-state migration coefficients (D_nssm_) for A and B concrete specimens are given in Figure 7.

The overall ranking of concrete quality in terms of chloride diffusion resistance for the tested concrete mixes made with ternary cements ranged from good to very good, as shown in Figure 7. Accordingly, the resistance to chloride penetration of concretes B and A made with ternary cements was very good and good, respectively.

The lowest chloride migration coefficient of 0.53 × 10^−12^ m^2^/s was determined in concrete B made with the ternary cement SA40VA25, while the highest, 2.97 × 10^−12^ m^2^/s was measured in concrete A made with the reference cement (CEM I).

The concrete mixes with 40% GGBFS (Type A) and 25% FA exhibited improved resistance to chloride ingress since the chloride diffusion and chloride migration coefficients were 2.89 × 10^−12^ m^2^/s and 0.53 × 10^−12^ m^2^/s, respectively, which indicates that their resistance to chloride diffusion is very good. This could be attributed to the synergic effect of both cement constituents working together (GGBFS and FA). Similar results (remarkably high resistance to chloride ingress) have been reported with ternary cements composed of 10% of silica fume and 20% of coal fly ash [43].

An important influence of the ground granulated blast-furnace slag on the compressive strength and chloride penetration resistance has been reported by Duży et al. [44]. This can partly be explained by the fact that there was a possible total porosity and effective porosity reduction due to the GGBFS addition. They found quasi-linear relationships between these parameters and the ground granulated blast-furnace slag content.

McNally and Sheils [45] tested concretes made with a mix of CEM II/A-L 42.5, CEM II/A-V 42.5, CEM III/B 32.5, and ground granulated blast-furnace slag, according to NT BUILD 492 at the age of 90 days. The values of water/binder were 0.45 and 0.55, corresponding to binder contents of 400 and 320 kg/m^3^, respectively. Ground granulated blast-furnace slag replacement levels were 0–70% for the various blends considered. The effective chloride diffusion coefficient ranged from 7.01 × 10^−12^ to 14.20 × 10^−12^ m^2^/s without ground granulated blast-furnace slag. With the incorporation of 50% and 70% ground granulated blast-furnace slag, the effective chloride diffusion coefficient is reduced by more than seven times as the slag content increases. For concretes without Supplementary Cementitious Materials (SCM), a water/cement ratio of 0.55 and cement content of 320 kg/m^3^, they found an effective chloride diffusion coefficient (NT BUILD 492) of 14.20 × 10^−12^ m^2^/s [45]. For concrete B, with a lower water/ratio and higher cement content (w/c = 0.5, 350 kg/m^3^), the effective chloride diffusion coefficient was 2.86 × 10^−12^ m^2^/s. The presence of 10% of coal fly ash in the cement results in an effective chloride diffusion coefficient of 9.14 × 10^−12^ m^2^/s. Furthermore, the incorporation of 50% or 70% results in effective chloride diffusion coefficients of 1.30 × 10^−12^ m^2^/s and 0.77 × 10^−12^ m^2^/s, respectively. Nevertheless, the coefficient determined in the cement with 10% coal fly ash and 70% ground granulated blast-furnace slag is higher (1.73 × 10^−12^ m^2^/s) than the one found in the cement with only 70% ground granulated blast-furnace slag (0.77 × 10^−12^ m^2^/s). Accordingly, the combined effect of cements containing ground granulated blast-furnace slag and coal fly ash in the cement is unclear. Similar conclusions have been reached in the present study. Furthermore, it has been reported that chloride migration coefficients determined at 28 and 182 days (NT BUILD 492) in concretes made of CEM III/B 42.5 N (66–70% slag), with water/cement ratio ranging from 0.40 to 0.55 and cement content from 320 to 400 kg/m^3^ ranged from 0.37 to 6.64 [46].

In general, the addition of pozzolanic cementing materials such as ground granulated blast-furnace slag and coal fly ash reduced the diffusion of chloride into concrete, as shown by a decrease recorded in the values of D_eff_ and D_nssm_ [31].

There was an important discrepancy between the results of the diffusion and migration coefficient, which was between 3 and 8 times lower for the migration coefficient than the diffusion one. It can be assumed that even the same concrete type has very disparate values of chloride penetration resistance when dissimilar testing procedures are utilized.

In this paper, the efficiency of the rapid chloride migration (RCM) test, performed in accordance with the test procedure specified in NT BUILD 492, in assessing the chloride penetration resistance of concrete made with ternary cements is presented. Overall, the rapid chloride migration (RCM) test is a reasonable indicator of the chloride ingress resistance of ternary concretes, having been able to distinguish concrete produced with different types of ternary cements. Nevertheless, this test may only be used by materials engineers to compare protective properties, related to the chloride penetration resistance, of tested concretes. Thus, it cannot be used to estimate the service life of reinforced concretes. This test procedure should not be used to determine the period of time after which the chloride ion content achieves the threshold level on the steel reinforcement surface of the reinforced concrete, since the chloride ion penetration throughout the concrete is described by a mechanism of ion flow controlled by diffusion.

**Figure 8:**
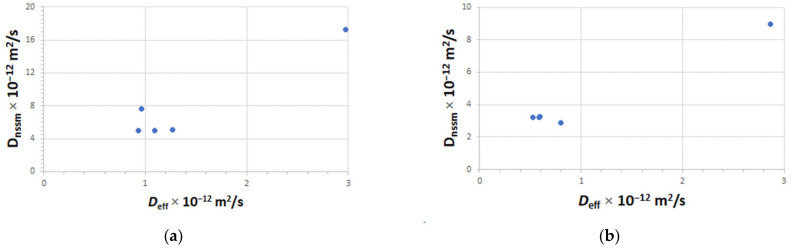
Chloride diffusion coefficient versus migration coefficient of concretes made with ternary cements: (**a**) A-Concrete; (**b**) B-Concrete.

Figure 8 shows that there is no significant relationship between the chloride diffusion coefficient and the migration coefficient of concretes A and B made with ternary cements. This means that each concrete made with different ternary cements belongs to different concrete families and, consequently, their results cannot be used to obtain a reliable correlation. In addition, it follows that the non-steady-state migration coefficient (D_nssm_), determined according to the NT BUILD 492 standard, characterizes the chloride ion penetration into concrete specimens when exposed to an electric field. However, this procedure does not provide the period of time after which the corrosion threshold of chloride ion concentration for corrosion initiation was reached at the surface of the embedded steel bar. Furthermore, the method described in the NT BUILD 492 standard may also be useful to compare the protective properties of concrete. Nevertheless, sometimes chloride ions migration through the concrete could be correlated with the chloride ion diffusion performance for the same concrete mix design. A reasonable correlation between the chloride diffusion from non-steady-state diffusion tests (D_nssm_) and the effective chloride diffusion (D_eff_) has been reported by some authors [47,48,49,50]. This suggests that, in some types of concretes, this accelerated method can be utilized for assessing concrete durability, for which strong correlations were found between the results achieved by performing experimental test methods according to NT BUILD 443 [16] and NT BUILD 492 [18].

In contrast, a lack of connection between natural chloride diffusion and chloride migration was also reported [51,52] when different types of concretes are assessed. Thus, it can only be used to compare qualitatively durability properties related to chloride-induced corrosion such as chloride penetration resistance.

### 3.4. Service Life Estimation

Commonly, the definition of the concrete requirements related with the durability of the reinforced concrete structures was by prescription of the 28-day compressive strength test and by the concrete composition (minimum cement content and maximum water/cement ratio) and, only in some cases, limits for some concrete characteristics were established, such as the chloride diffusion coefficient among others.

The critical chloride content can be defined as the chloride content required for passivation of the steel or the chloride content associated with acceptable deterioration of the reinforced concrete structure. The critical chloride content normally is expressed as total chloride ion content relative to the weight of the Portland cement, i.e., C_x,t_. Numerous publications in connection with the critical chloride content can be found in the literature, whereby a set of values ranging from 0.05% to 8% are given. Most of these values are between 0.1% and 1%. 

**Figure 9:**
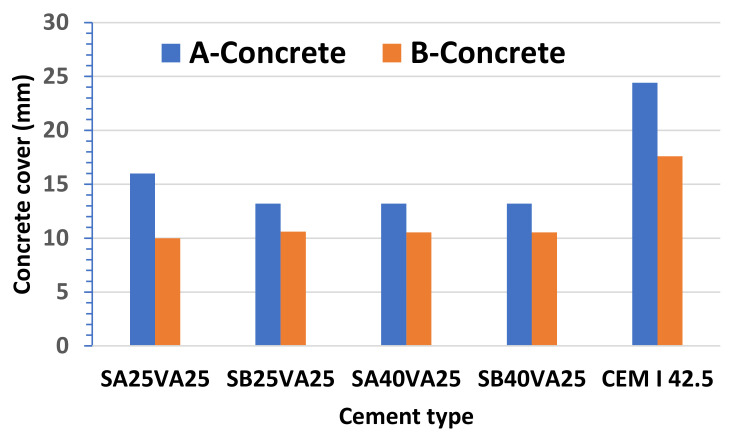
Minimum cover to ensure 50 years of service life of reinforced concrete structures by using concretes made with ternary cements (X, mm).

A large number of critical chloride content or chloride threshold values can be found in the published literature. Most of them are based on Portland cement. In contrast, recent studies on other types of cements have often provided conflicting results. For instance, lower chloride threshold values for ground granulated blast-furnace slag containing concrete in comparison with CEM I concrete have been reported in some studies [53], whereas other studies showed the opposite [54]. It has been a widespread practice in Europe and in North America to limit the chloride content to about 0.4% by weight of cement [55], while the fib model code for service life design defines a critical chloride content mean value of 0.6% [56]. Accordingly, there is a need for reliable chloride threshold values. Currently, a standardised test method to determine the critical chloride does not exist. For the present study, we have considered an average critical chloride content of 0.55%. In addition, we have chosen a chloride ion concentration at the concrete surface, C_s_, of 0.6% to conduct the present study. Figure 9 shows the estimated cover to ensure a 50 years of service life of reinforced concrete structures by using concretes made with ternary cements collected in Table 2. The minimum required concrete cover thickness for reinforcement normally ranges between 20 and 55 mm depending on the environmental conditions throughout the reinforced concrete structure’s service life. The larger concrete cover thickness is found in the concretes made with CEM I, i.e., 24.4 mm and 17.6 mm for concretes A and B, respectively. In contrast, it was found that smaller concrete cover thickness is required for concretes made with ternary cements to guarantee a service life of 50 years, ranging from 10.5 mm to 16 mm. Since all of them have lower values than the recommended minimum Structural Class S1 [57], it may be said that these ternary-cement-concretes are very suitable for use in exposure classes related to the corrosion induced by chlorides.

Table 6 shows the values of minimum cover requirements regarding durability for reinforcement steel according to EN 1992-1-1 [57] for exposure classes related to environmental conditions. The recommended Structural Class (design working life of 50 years) is S4 for the indicative concrete strengths given in EN 1992-1-1 [57]. Concrete cover protects the reinforcement from chloride-induced corrosion initiation. Then, the minimum concrete cover depth required to be provided for corrosion against chlorides ingress according to Eurocode 2 is given in Table 6, where the corrosion induced by chloride exposure classes (XD and XS) is defined.

Since the objective of this study is to evaluate the achievement of GGBFS-FA cements regarding their chloride penetration, the chloride diffusion coefficient of ternary cement concretes was utilized to assess their potential for improvement. Consequently, the minimum cover of concrete, which is required to prevent corrosion induced by chlorides, was taken as reference figure.

Finally, these results will be useful to material and civil engineers in designing reinforced concrete made with ternary cements. In any case, the service life of a building depends mainly on its chief structural materials and the environment in which it is placed.

## 4. Conclusions

The following conclusions can be drawn based on the experimental data and analysis of the diffusion and migration coefficients conducted in the present study.

- No relationship between diffusion and migration coefficients was found since the tested concretes belong to different “concrete families”, i.e., each ternary cement originates a peculiar concrete type.
- As expected, the supplementary cementing materials (25% FA and 25% or 40% GGBFS) in the cement improved the chloride resistance of concrete, which was evidenced by very low diffusion and migration coefficients (2–5 times less than that in CEM I concrete). Furthermore, the performance of 40% ground granulated blast-furnace slag cement concrete (SB40VA25) is better than that of 25% GGBFS and 25% FA (SB25VA25) cement concretes.
- The lowest chloride migration coefficient was measured in the concrete prepared with SB40VA25 which is 0.59 × 10^−12^ m^2^/s (concrete B), while the highest chloride migration coefficient was measured in SB25VA25 ternary cement concrete made with 25% GGBFS and 25% FA which is 1.27 × 10^−12^ m^2^/s. Accordingly, the highest chloride migration coefficient value doubles the value of the lowest chloride migration coefficient for the ternary cement concretes. The resistance to chloride ingress in the concrete of specimens SB40VA25 and SB25VA25 is high and moderate, respectively, as per the NT BUILD 492 classification.
- The diffusion coefficient determined as laid down in the international standard NT BUILD 443 adequately describes the diffusion process of chloride ions through the tested concrete. Therefore, the chloride diffusion coefficient determined with this method can be used for service life estimations. Similar values for the diffusion coefficient were found in concretes made with ternary cements SA40VA25 and SB40VA25.

All the above results could be taken into account by concrete designers to build reinforced concrete structures made of concrete containing ternary cements made with ground granulated blast-furnace slag (GGBFS) and coal fly ash (FA).

## Figures and Tables

**Table 1:** Chemical characteristics of the CEM I 42.5 R, GGBFS, and siliceous fly ash, FA (%).

Constituent	SiO_2_	Al_2_O_3_	Fe_2_O_3_	CaO	MgO	SO_3_	Na_2_O	K_2_O	LOI	IR ^1^	Cl^−^
CEM I 42.5 R	20.51	4.30	3.01	60.38	3.61	3.14	0.16	0.81	2.78	1.44	0.05
GGBFS	35.96	10.61	0.40	42.89	7.10	2.02	0.30	0.46	0.00	–	–
FA	53.79	19.54	10.20	4.44	1.83	0.84	2.03	1.83	1.73	17.41	–

^1^ Measured by Na_2_CO_3_ method described in EN 196-2.

**Table 2:** Ternary cement mix design.

Denomination	CEM I (%)	GGBFS (%)	FA (%)	GGBFS—Fineness (cm^2^/g)
CEM I	100	0	0	–
SA25VA25	50	25	25	3489
SA40VA25	35	40	25	3489
SB25VA25	50	25	25	4630
SB40VA25	35	40	25	4630

**Table 3:** Concrete mix design (kg/m^3^).

Concrete	Cement	Sand	Gravel	Water	Additive
A (kg/m^3^)	250	880	1100	172	5.0
B (kg/m^3^)	350	840	1100	172	5.0

**Table 4:** Cement constituents for each concrete, i.e., A and B (kg/m^3^).

Denomination	A—Concrete				B—Concrete			
	CEM I	SA	SB	VA	CEM I	SA	SB	VA
CEM I 42.5 R	250				350			
SA25VA25	125	62.5		62.5	175	87.5		87.5
SB25VA25	125		62.5	62.5	175		87.5	87.5
SA40VA25	87.5	100		62.5	122.5	140		87.5
SB40VA25	87.5		100	62.5	122.5		140	87.5

**Table 5:** Chloride diffusion coefficient (D_eff_ × 10^−12^ m^2^/s) determined after 90 days of testing time.

Cement Type	A-Concrete	B-Concrete
SA25VA25	7.64	2.89
SB25VA25	5.06	3.26
SA40VA25	4.98	3.21
SB40VA25	4.98	3.21
CEM I 42.5	17.3	8.99

**Table 6:** Minimum cover depth of concrete (mm) for corrosion induced by chlorides.

Environmental Requirement for Minimum Concrete Cover Depth (mm)	Structural Class					
**Exposure Class:** **Corrosion induced by chlorides**	**S1**	**S2**	**S3**	**S4**	**S5**	**S6**
XD1-Moderate humidity	20	25	30	35	40	45
XD2-Wet, rarely dry	25	30	35	40	45	50
XD3-Cyclic wet and dry	30	35	40	45	50	55
**Exposure Class:** **Corrosion induced by chlorides from sea water**	**S1**	**S2**	**S3**	**S4**	**S5**	**S6**
XS1-Exposed to airborne salt but not in direct contact with sea water	20	25	30	35	40	45
XS2-Permanently submerged	25	30	35	40	45	50
XS3-Tidal, splash and spray zones	30	35	40	45	50	55

## Data Availability

Data are contained within the article.
